# Genetic and Environmental Factors of Social Adaptation in Schizophrenia: The Role of OXTR, AGER, and Adverse Childhood Experiences

**DOI:** 10.1192/j.eurpsy.2026.11267

**Published:** 2026-07-31

**Authors:** V. A. Mikhailova, T. V. Lezheiko, V. Golimbet, V. V. Plakunova

**Affiliations:** Clinical Genetics Laboratory, Mental Health Research Center, Moscow, Russian Federation

## Abstract

**Introduction:**

Social functioning (SF) is a critical determinant of quality of life and prognosis in schizophrenia. Impairments in social adaptation may manifest even in the premorbid stage, suggesting a genetic basis. The oxytocin receptor gene (OXTR) and the receptor for advanced glycation end-products gene (AGER) have been implicated in social behavior regulation, including oxytocin transport across the blood–brain barrier. Adverse childhood experiences (ACEs) are strong environmental modifiers of schizophrenia outcomes.

**Objectives:**

To assess the contribution of OXTR rs1042778 and AGER rs1800625 polymorphisms to social dysfunction in schizophrenia, accounting for the influence of ACEs. We hypothesized that genetic variants in OXTR and AGER interact with ACEs to worsen SF outcomes.

**Methods:**

The study included 507 patients (65.9% female, mean age 36.2±12.6 years, mean illness duration 12.2±10.4 years) diagnosed with schizophrenia spectrum disorders (ICD-10 F20.x, F25.x). SF was assessed with the Personal and Social Performance (PSP) scale; symptoms were rated with PANSS. Genotyping: OXTR rs1042778 by HRM, AGER rs1800625 from genome-wide SNP arrays. ACEs were evaluated from medical records. Statistical analyses included ANCOVA with sex and illness duration as covariates; multiple testing correction by Benjamini–Hochberg FDR.

**Results:**

A significant association was found between AGER rs1800625 and PSP disturbing/aggressive behavior (F=10.424, p=0.002, pBH=0.024). A joint effect of OXTR rs1042778 and AGER rs1800625 was observed on PSP socially useful activities (F=5.286, p=0.024). A trend-level interaction with ACEs was noted for interpersonal relationships (F=5.060, p=0.027; n.s. after FDR). OXTR rs1042778 showed nominal association with self-care (F=4.534, p=0.036; n.s. after FDR).

**Conclusions:**

This study demonstrates, for the first time, an association of AGER rs1800625 with impaired SF in schizophrenia, particularly with disturbing/aggressive behavior. Combined effects of OXTR and AGER polymorphisms and the modulatory role of ACEs highlight the interplay of oxytocinergic and inflammatory pathways in shaping social dysfunction. These findings may contribute to future personalized approaches targeting social outcomes in schizophrenia.

**Disclosure of Interest:**

None Declared

